# Association Between COVID-19 Outcomes and Patient Characteristics: A Study in an Inner-City Community Hospital

**DOI:** 10.7759/cureus.17255

**Published:** 2021-08-17

**Authors:** Marcos Almonte, Xiu Ying Au, Mustafa Ali, Nafiisah Rajabalee, Syed Hasan, Temesgen Shibre, Yiting Li, Adam C Kaplan

**Affiliations:** 1 Internal Medicine, Jersey Shore University Medical Center/Saint Francis Medical Center Program, Trenton, USA; 2 Internal Medicine, Saint Francis Medical Center, Seton Hall University Hackensack Meridian School of Medicine, Trenton, USA

**Keywords:** lymphopenia, thrombocytopenia, charlson comorbidity index, covid-19, sars-cov-2

## Abstract

Background

It has been shown that certain hematological conditions, such as lymphopenia and thrombocytopenia, are associated with increased severity and mortality from severe acute respiratory syndrome coronavirus 2 (SARS-CoV-2) infection. However, a majority of the previous data came from large institutional centers with high academic output. We aimed to explore the association between patient’s characteristics, hematological parameters, and outcomes in admitted persons with coronavirus disease 2019 (COVID-19) at our community hospital in an inner city.

Methods

This study is a retrospective chart review designed to evaluate the potential associations between demographic and clinical characteristics of our patient population and their outcomes when testing positive for SARS-CoV-2. The study population included patients hospitalized in the Saint Francis Medical Center from January 2020 to September 2020. This pilot study included 50 out of the 275 hospitalized patients with a confirmed SARS-CoV-2 infection during this timeframe. Data collection from the patient’s chart included age, sex, comorbidities, admission complete blood cell count, and use of Remdesivir, steroids, and plasma. The primary outcome was all-cause in-hospital mortality. Secondary outcomes included the need for mechanical ventilation and ICU admission.

Results

In this pilot study, there was an overall mortality rate of 32% (16 out of 50 patients). Charlson Comorbidity Index (CCI) of 3 points or above was present in 87.5% of the patients in the mortality group versus 41.1% in patients who survived (p = 0.0021). There was no statistically significant difference in mortality between males and females after adjusting for other variables with an odds ratio (OR) of 0.19 (95% CI 0.02-1.80, p = 0.09). There were no statically significant differences in mortality between Caucasians, non-Hispanic, Black, and Latinx patients (p = 0.466). Admission platelets were lower in the mortality group with a mean of 157.7 ± 43.23 (Thou/ul) versus 250.06 ± 93.95 (Thou/ul) in the survivors (p = 0.0005). Admission white blood cell count in the mortality group was lower than the survivor group with an average of 5.93 ± 2.58 (Thou/ul) versus 9.3 ± 4.14 (Thou/ul) (p = 0.0039), respectively. The plasma D-dimer level of 3 mg/L fibrinogen equivalent unit (FEU) or higher was associated with increased mortality. There was no association of C-reactive protein (CRP) with mortality (p = 0.93 and p = 0.54, respectively); however, the CRP level revealed an association with ICU admission (p = 0.03). The use of steroids, Remdesivir, and plasma did not have a statistically significant effect on mortality, ICU admission, or sepsis in our study.

Conclusion

In this study, older age, higher CCI, and plasma D-dimer level of 3 mg/L FEU or higher were associated with higher mortality among COVID-19 patients. White blood cell count and platelet count were significantly lower in the mortality group in comparison to the survivor group. However, there was no statistical difference in lymphocyte count between the mortality group and the survivor group. COVID-19 patients with thrombocytopenia or serum CRP level of 15 mg/dL or higher were more likely to be admitted to ICU.

## Introduction

The severe acute respiratory syndrome coronavirus 2 (SARS-CoV-2) pandemic shook the entire world. Our healthcare systems are still strained as the global death toll peaks at more than 2 million to date. Several co-morbidities like diabetes mellitus, hypertension, cardiovascular diseases, pulmonary diseases, obstructive sleep apnea, and access inequities were observed to be risk factors for worse outcomes [1,2]. At our community hospital in the suburban US, we aimed to explore the association between our coronavirus disease 2019 (COVID-19) patients' characteristics and outcomes. Our analysis, similar to worldwide observations, demonstrated that that older age and higher Charlson Comorbidity Index (CCI) were linked to higher mortality. We further noted that our patients who unfortunately died from COVID-19 had lower white blood cells and platelet counts at admission. In order to generalize our findings into more clinically significant data, we look forward to liaising with other community hospitals. Combined data can certainly help us manage patients with evidence-based medicine.

## Materials and methods

We performed a retrospective analysis of adult patients hospitalized with COVID-19 at Saint Francis Medical Center between January 2020 to September 2020. The study protocol was approved by the institutional review board at Saint Francis Medical Center. Inpatient adults who tested positive for SARS-CoV-2 polymerase chain reaction (PCR) on nasopharyngeal swabs, during the index admission were included. This study included 50 randomized patients out of the 275 hospitalized patients with confirmed SARS-CoV-2 infection. Patients were picked for inclusion utilizing a randomized numbers generator. Data were collected on baseline registration characteristics, comorbidities, and admission complete blood cell count and differentials. A Charlson Comorbidity Index, which predicts 10-year survival in comorbid patients, was calculated for all included participants. In addition, the use of steroids or antibiotics prior to admission or during hospitalization was recorded. Chart diagnosis, as well as hospital coding diagnosis (specifically for the diagnosis of sepsis), were recorded.

The primary outcome was all-cause in-hospital mortality. Secondary outcomes included the need for mechanical ventilation, sepsis, and ICU admission. We utilized chart reviews and hospital billing to obtain our outcome data. For normally distributed continuous data, comparisons between independent groups were made using Student’s t-tests. Categorical variables were compared between groups using the chi-square test to search for possible statistical significance. All analyses were performed by using SAS version 9.4 (SAS Institute, Cary, North Carolina).

## Results

During the study window, 50 patients who were admitted with COVID-19 were randomly selected. Table 1 shows the demographic characteristics. Our patient population’s insurance status was also reported. Of the patients, 32% carried private/commercial insurance, 16% had Medicaid, 26% were prisoners, and 2% had charity care or self-pay. Of those who carried Medicare, 10% had Medicare A+B and 80% had Medicare + Medicaid. The mean age was 60.2 ± 18.7 years. Among 50 patients, 16 (32%) had died. The mean age of patients who died was 74 ± 9 years, which was older compared to the mean age of those who survived (53 ± 18 years with a p-value of <0.001). Among patients who died, 62.5% were female. There was no statistically significant difference in BMI of patients who survived versus those who died (p = 0.131). Non-Hispanic white patients comprised 37.5% of patients who died, while non-Hispanic black patients and Hispanic patients each comprised 25.0% of the same patient group. Of the patients who died from COVID-19, 87.5% had a Charlson Comorbidity Index (CCI) of 3 or more.

**Table 1:** Characteristics of patients at baseline, overall, and comparison between patients who survived versus patients who died.

Characteristic	Overall (n = 50)	Survived (n = 34)	Died (n = 16)	p-value
Mean age - year	60.2 ± 18.7	53 ± 18	74 ± 9	<0.001
Gender - n (%)				
Male	32/50 (64.0%)	26/34 (76.5%)	6/16 (37.5%)	
Female	18/50 (36.0%)	8/34 (23.5%)	10/16 (62.5%)	
Body mass index (BMI)	29.5 ± 8.5	28.3 ± 8.6	32.1 ± 7.9	0.131
Race - n (%)				
White, non-Hispanic	12/50 (24.0%)	6/34 (17.6%)	6/16 (37.5%)	
Black, non-Hispanic	17/50 (34.0%)	13/34 (38.2%)	4/16 (25.0%)	
Hispanic	15/50 (30.0%)	11/34 (32.4%)	4/16 (25.0%)	
Other	6/50 (12.0%)	4/34 (11.8%)	2/16 (12.5%)	
Charlson Comorbidity Index (CCI) - n (%)				
0	9/50 (18.0%)	9/34 (26.5%)	0/16 (0.0%)	
1-2	13/50 (26.0%)	11/34 (32.3%)	2/16 (12.5%)	
≥3	28/50 (56.0%)	14/34 (41.2%)	14/16 (87.5%)	

Table 2 shows hematological values at admission and Table 3 shows values of serum inflammatory markers at admission. The mean white blood cell count at admission was 5.9 in patients who died, which was lower compared to 9.3 in those who survived (p = 0.003). The mean platelet count on admission was 157 in patients who died, which was also lower compared to those who survived (250, p < 0.001). The following laboratory values at admission, such as hemoglobin level (p = 0.947), absolute neutrophil count (p = 0.157), absolute lymphocyte count (p = 0.222), absolute eosinophil count (p = 0.249), serum C-reactive protein (CRP) level (p = 0.292), and plasma D-dimer level (p = 0.220), were not statistically different between patients who died and who survived.

**Table 2:** Hematological values at admission, overall, and comparison between patients who survived versus patients who died.

Hematological values	Overall (n = 50) (Mean, 95% CI)	Survived (n = 34) (Mean, 95% CI)	Died (n = 16) (Mean, 95% CI)	p-value
Hemoglobin level, g/dL	12.7 (12.1-13.3)	12.7 (11.8-13.6)	12.7 (11.9-13.5)	0.947
Platelet count, x 10^3^ cells/µL	220 (195-246)	250 (217-283)	157 (134-180)	<0.001
White blood cell count, x 10^3^ cells/µL	8.2 (7.1-9.3)	9.3 (7.9-10.8)	5.9 (4.5-7.3)	0.003
Absolute neutrophil count, x 10^3^ cells/µL	7.3 (4.6-10.1)	8.7 (4.6-12.8)	4.4 (3.2-10.2)	0.157
Absolute lymphocyte count, x 10^3^ cells/µL	2.0 (0.8-3.2)	2.5 (0.8-4.2)	0.9 (0.8-1.0)	0.222
Absolute eosinophil count, x 10^3^ cells/µL	0.1 (0-0.3)	0.2 (0-0.5)	0 (0)	0.249

**Table 3:** The values of serum inflammatory markers at admission, overall, and comparison between patients who survived versus patients who died. FEU = fibrinogen equivalent unit.

Values of Inflammatory markers	Overall (n = 50)		Survived (n = 34)		Died (n = 16)		p-value
	Available data, Mean (95% CI)	Missing data, n (%)	Available data, Mean (95% CI)	Missing data, n (%)	Available data, Mean (95% CI)	Missing data, n (%)	
Serum C-reactive protein level, mg/dL	12.5 (10.4-14.7)	11/50 (22.0%)	11.7 (8.7-14.6)	8/34 (23.5%)	14.4 (12.4-16.4)	3/16 (18.8%)	0.292
Plasma D-dimer level, mg/L FEU	1.75 (1.27-2.22)	9/50 (18.0%)	2.0 (0.7-3.2)	7/34 (20.6%)	3.7 (1.2-6.3)	2/16 (12.5%)	0.220

Table 4 shows factors associated with mortality, ICU admission, and intubation in adults hospitalized with COVID-19. CCI of 3 points or more and plasma D-dimer level of 3 mg/L fibrinogen equivalent unit (FEU) or higher were strongly associated with the death of COVID-19 patients. Patients who required ICU admissions were more likely to have a platelet count of less than 150,000 cells/µL. Those who were admitted to ICU were also more likely to have a serum CRP level of 15 mg/dL or higher. There were no significant associations identified between intubation and the following factors: female gender, non-white races, CCI of 3 or higher, platelet count of less than 150,000 cells/µL, white blood cell count of less than 4,500 cells/µL, serum CRP of 15 mg/dL or higher, and plasma D-dimer of 3 mg/L FEU or higher.

**Table 4:** Factors associated with mortality, ICU admission, and intubation in adults hospitalized with COVID-19. CCI = Charlson Comorbidity Index; CRP = C-reactive protein; FEU = fibrinogen equivalent unit.

Factors	Mortality		ICU admission		Intubation	
	Adjusted odds ratio (95% CI)	p-value	Adjusted odds ratio (95% CI)	p-value	Adjusted odds ratio (95% CI)	p-value
Female gender	5.15 (0.43-61.44)	0.195	3.49 (0.24-50.78)	0.360	4.73 (0.20-109.76)	0.333
Non-white races	0.03 (0.00-0.64)	0.025	1.02 (0.07-16.07)	0.988	0.38 (0.02-9.29)	0.552
CCI ≥3	134.54 (4.21-4298.52)	0.006	15.37 (0.75-317.42)	0.077	8.79 (0.38-202.95)	0.175
Platelet count <150, x 10^3^ cells/µL	6.63 (0.68-64.47)	0.103	18.07 (1.11-294.57)	0.042	5.82 (0.31-107.95)	0.237
White blood cell count <4.5, x 10^3^ cells/µL	13.91 (0.07-2890.58)	0.334	5.78 (0.18-182.49)	0.319	13.38 (0.32-558.39)	0.173
Serum CRP ≥15 mg/dL	9.47 (0.90-99.90)	0.062	45.01 (1.59-1272.47)	0.026	66.39 (0.96-4615.52)	0.053
Plasma D-dimer ≥3 mg/L FEU	147.60 (2.92-7461.94)	0.013	24.14 (0.45-1293.07)	0.117	50.46 (0.42-6041.47)	0.108

Odds ratios were adjusted for the female gender, non-white races, Charlson Comorbidity Index (CCI) ≥3, platelet count <150,000 cells/µL, white blood cell count <4,500 cells/µL, serum CRP level ≥15 mg/dL, and plasma D-dimer ≥3 mg/L FEU.

## Discussion

In this study, we found an association between lower platelets and those with higher mortality, which is consistent with the literature [3-5]; we also found an association between lower leukocyte count at admission and increased mortality secondary to COVID-19, which differs from some of the currently available studies [4], and there were no statistically significant associations between admission absolute eosinophil count, hemoglobin, lymphocyte count, or neutrophil count and the risk of death from COVID-19 infection.

Critical COVID-19 infection has been associated with the involvement of multiple organ systems and its natural course has been linked to the development of refractory hypoxia, acute respiratory distress syndrome (ARDS), multiorgan failure, and coagulopathy [6,7]; thrombocytopenia has been consistently reported among the patients with a severe disease, which is probably linked to the disseminated intravenous coagulation (DIC) observed in these patients [5]. At this moment, the pathophysiology of the disease is not well understood, and more studies are necessary to guide proper management to prevent the coagulopathy that affects COVID-19 patients and recognizing thrombocytopenia as an early sign of a potential coagulopathy might help guide the decision of using prophylactic versus therapeutic anticoagulation in patients with a critical illness.

The patient’s age was an important factor in determining the risk of mortality, in our study, higher age (74 ± 9 years) was associated with worse outcomes, which is consistent with general knowledge of the disease; we did not observe higher mortality in males versus females, which has been clearly documented in the medical literature, including reports from the CDC [8], and other available studies [9]. This finding may be due to specific socio-economic and demographic differences between our patient population and the population previously studied. Additionally, the number of female patients compared with male patients in our study population may have biased our results.

Higher mortality, ICU admissions, and intubations were observed among the patients with a D-dimer >3 mg/L FEU and CRP >15mg/dL. Increased D-dimer and CRP have been consistently reported in severe cases of COVID-19 infection [10], and are linked to the increased inflammation and coagulopathy in patients with COVID-19; a D-dimer >3mg/L FEU in COVID-19 patients has been previously associated with an increased risk of a thromboembolic event despite the use of prophylactic anticoagulation [11], which might also be tightly linked to microthrombosis of the pulmonary vasculature, but its use as a single predictor is limited [12]. Using the available studies which show increased risk of severe disease with a higher D-dimer and our described association between lower platelets and worse outcomes, further studies should aim to identify if there is any prognostic signal to help difference when to use prophylactic or therapeutic doses of anticoagulation in patients with lower platelets and a D-dimer >3 mg/L FEU.

A Charlson Comorbidity Index >3 was associated with higher mortality and has proven itself useful as a prognostic tool for patients with COVID-19 by itself and in association with other variables [13,14]. Its use based on the congruence of data between small and large institutions should be promoted based on our interpretation of the data.

Our study has several limitations, including but not limited to the small patient population, differences between the amount of male and female patients, limited analysis of the therapies used, including dose and duration, and the changing treatment algorithm since our patient’s recruitment and analysis. However, most of our primary and secondary outcomes are consistent with available literature and we believe that further analysis of thrombocytopenia and elevated D-dimer may contribute to the current grey zone of how to utilize anticoagulation in a more nuanced, evidence-based manner. Lastly, documenting the demographics, trends, outcomes, and findings for small community hospitals is important as the populations that tend to have the highest mortality are often treated by clinicians working in safety-net hospitals without the vast resources of an academic medical center.

## Conclusions

In conclusion, older age, higher CCI, and plasma D-dimer level of 3 mg/L FEU or higher were associated with higher mortality among COVID-19 patients. White blood cell count and platelet count were significantly lower in the mortality group in comparison to the survivor group. However, there was no statistical difference in lymphocyte count between the mortality group and the survivor group. COVID-19 patients with thrombocytopenia or serum CRP level of 15 mg/dL or higher were more likely to be admitted to ICU.
